# Spin effects on transport and zero-bias anomaly in a hybrid Majorana wire-quantum dot system

**DOI:** 10.1038/s41598-023-44254-9

**Published:** 2023-10-12

**Authors:** Alexandre Huguet, Kacper Wrześniewski, Ireneusz Weymann

**Affiliations:** 1 Institute of Spintronics and Quantum Information, Faculty of Physics, Adam Mickiewicz University, Uniwersytetu Poznańskiego 2, 61-614 Poznań, Poland; 2 Université de Rennes 1, Campus de Beaulieu, F-35000 Rennes, France

**Keywords:** Topological defects, Spintronics, Quantum dots, Nanowires

## Abstract

We examine the impact of spin effects on the nonequilibrium transport properties of a nanowire hosting Majorana zero-energy modes at its ends, coupled to a quantum dot junction with ferromagnetic leads. Using the real-time diagrammatic technique, we determine the current, differential conductance and current cross-correlations in the nonlinear response regime. We also explore transport in different magnetic configurations of the system, which can be quantified by the tunnel magnetoresistance. We show that the presence of Majorana quasiparticles gives rise to unique features in all spin-resolved transport characteristics, in particular, to zero-bias anomaly, negative differential conductance, negative tunnel magnetoresistance, and it is also reflected in the current cross-correlations. Moreover, we study the dependence of the zero-bias anomaly on various system parameters and demonstrate its dependence on the magnetic configuration of the system as well as on the degree of spin polarization in the leads. A highly nontrivial behavior is also found for the tunnel magnetoresistance, which exhibits regions of enhanced or negative values—new features resulting from the coupling to Majorana wire.

## Introduction

Topological states of matter are in the center of both theoretical and experimental research worldwide^1–3^, which is triggered by their unique features associated with protection, stemming from the system’s topology, against decoherence and noise^4^. In this respect, Majorana zero-energy modes (MZM) that appear at the ends of a quantum wire (Majorana wire), play a particularly important role^5,6^. Such modes constitute condensed-matter realizations of long-searched Majorana fermions^7^, and have been attracting a great attention due to possible applications in topological quantum computing^8,9^. In fact, MZMs exhibit non-Abelian statistical properties, which are forecast as a key feature for future topologically protected and decoherence-free qubits^10,11^. Conceptually, a Majorana wire can be realized in topological phase of a Kitaev chain, i.e. a tight-binding chain with triplet pairing^8^. Such chains can be implemented e.g. in topological superconducting nanowires with strong spin-orbit interaction subject to magnetic field^12,13^, or in one-dimensional spiral magnetization textures, where Majorana modes emerge in the absence of magnetic field^14^. The recent progress in experimental techniques and fabrication of Kitaev chains makes nowadays the investigations of MZMs even more appealing^15–17^.

The presence of Majorana modes gives rise to unique transport properties, with the zero-bias anomaly in the differential conductance of the device being one of the most important features^18–21^. An interesting twist arises when one explores transport properties of hybrid structures, in which Majorana wire is additionally coupled to zero-dimensional systems, such as quantum dots (QDs)^22–34^. Transport characteristics of such hybrid quantum dots reveal then further distinctive properties, including fractional values of conductance, resulting from the leakage of Majorana modes into the dots and their half-fermionic nature^29,35–37^. We note that such half-fermionic Majorana nature can be also revealed in thermodynamic properties of the system, namely through the entropy^38–40^, which provides further insight into the system’s behavior^41–43^. Here, however, we focus mainly on the transport spectroscopy, which provides a convenient tool to distinguish and validate the MZM presence in the system. In addition, further insight can be obtained from the studies of the shot noise and, in particular, the current cross-correlations^44–47^. Such cross-correlations in hybrid quantum dot-Majorana systems have already been studied in the case of nonmagnetic contacts^48–52^.

Here, we extend those considerations to the case of spin-resolved tunneling caused by attaching the quantum dot to two separate ferromagnetic electrodes. We note that the considered setup is very similar to Cooper pair splitter geometries^53^, in which ferromagnetic leads have been shown to be crucial in detecting the entanglement between split Cooper pair electrons^54,55^. Although, here, instead of conventional superconductor, one has a nanowire with Majorana-like quasiparticles at its ends, the splitter geometry is still important, as the arrangement of magnetic moments of the electrodes provides additional tool to explore the signatures of leakage of MZMs into the quantum dot system.

In our considerations the focus is on the weak coupling regime between the ferromagnetic leads and the quantum dot, while the coupling to Majorana wire is arbitrary. In particular, we determine the transport characteristics, such as the current, differential conductance, current cross-correlations, and tunnel magnetoresistance, for various parameters of the system in the nonlinear response regime. The calculations are performed with the aid of the real-time diagrammatic technique in the lowest-order expansion with respect to the coupling to normal leads^56–58^. Special attention is paid to the effects of spin-resolved tunneling on the transport behavior and, especially, on the zero-bias anomaly associated with the presence of MZM, which we explore for two different cases of biasing the system. For symmetrically biased device, we examine the current flowing to the Majorana wire, finding asymmetric behavior of the transport characteristics with respect to the bias voltage, with inverted tunnel magnetoresistance (TMR) in most of the transport regimes. On the other hand, for asymmetrically biased device, the focus is on transport to one of the ferromagnetic leads. In this case we find modified Coulomb diamonds, with zero-bias signatures of MZM and greatly modified TMR as compared to the bare quantum dot case. We also explore the impact of spin-resolved tunneling on zero-bias anomaly in the differential conductance and show that its magnitude strongly depends on the degree of spin polarization of the leads. Finally, we determine the cross-correlations between the currents flowing to the normal ferromagnetic electrodes. Our study provides a comprehensive understanding of spin effects on the nonlinear transport properties of Majorana-quantum dot systems with ferromagnetic leads and the zero-bias anomaly due to the presence of Majorana quasiparticles. We believe that our work shall foster further investigations of hybrid quantum dot-Majorana systems.

## Results

### Model and Hamiltonian

**Figure 1:**
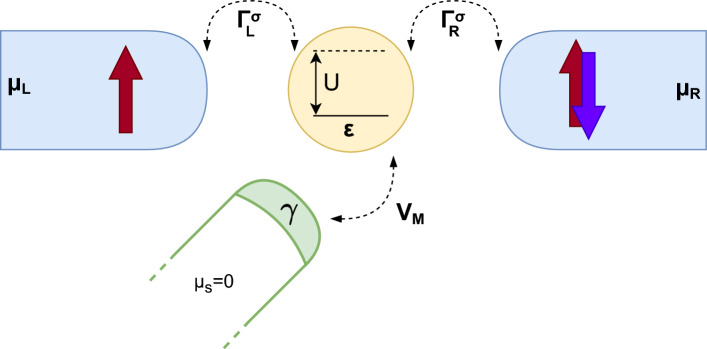
The schematic of the considered hybrid quantum dot-Majorana nanowire system. The quantum dot is coupled by $V_M$ to Majorana zero-energy mode at the end of the wire, described by $\gamma$, and weakly coupled to two ferromagnetic leads by the coupling constants $\Gamma _{\alpha }^\sigma$. The energy of orbital level of quantum dot is denoted by $\varepsilon$, while *U* stands for the Coulomb correlations. We consider two different ways the system is biased: *symmetric* one when bias voltage is applied between the leads ($\mu _L = \mu _R = eV$) and the Majorana wire and *asymmetric* one when $\mu _L = -\mu _R = eV/2$. In all cases the Majorana wire is assumed to be grounded ($\mu _S = 0$). The magnetic moments of the leads are assumed to form either parallel or antiparallel magnetic configuration, as indicated.

The considered hybrid system consists of a single-level quantum dot weakly coupled to two ferromagnetic leads and arbitrarily attached to a quantum wire hosting Majorana zero-energy modes at its ends, as shown in Fig. 1. It is further assumed that the quantization axis of MZM coincides with magnetizations of external ferromagnetic leads. Moreover, we assume that the magnetic moments of electrodes are collinear and can be found either in the parallel (P) or antiparallel (AP) magnetic configuration. We note that this assumption implies that the topological phase of Majorana wire and the way it couples to the quantum dot is not affected by the change of magnetic configuration of the system. Such situation could be achieved by using e.g. local magnetic fields or ferromagnetic split-gates^59,60^. The considered system can be described by an extended Anderson impurity Hamiltonian of the following general form

1 $$\begin{aligned} H = H_\text{Leads} + H_\text{Tun} + H_\mathrm{QD-Maj}. \end{aligned}$$

The leads are modeled as noninteracting quasiparticles, $H_\text{Leads} = \sum _{\alpha =L,R}\sum _{\textbf{k}\sigma } \varepsilon _{\alpha \textbf{k}\sigma } c^{\dag }_{\alpha \textbf{k}\sigma } c_{\alpha \textbf{k}\sigma }$. Here, $\varepsilon _{\alpha \textbf{k}\sigma }$ and $c^{\dag }_{\alpha \textbf{k}\sigma }$ denote the single-particle energy and the fermionic creation operator, respectively, of an electron with spin $\sigma$ momentum $\textbf{k}$ in the left ($\alpha =L$) or right ($\alpha =R$) lead. The coupling between the reservoirs and the quantum dot is described by the tunneling Hamiltonian, $H_\text{Tun} = \sum _{\alpha =L,R} \sum _{\textbf{k}\sigma } (t_{\alpha \textbf{k}\sigma } c^{\dag }_{\alpha \textbf{k}\sigma } d_{\sigma } + H.c.)$, with $t_{\alpha \textbf{k}\sigma }$ being the relevant tunneling matrix elements and $d^{\dag }_{\sigma }$ denoting the intra-dot fermionic creation operator for an electron of spin $\sigma$. The coupling of the dot to external leads gives rise to the broadening of quantum dot energy levels given by $\Gamma = \sum _{\alpha \sigma } \Gamma ^\sigma _\alpha$, where $\Gamma ^\sigma _\alpha$ denotes the coupling strength to the lead $\alpha$ for spin $\sigma$. Assuming the matrix elements $t_{\alpha \textbf{k}\sigma }$ to be momentum-independent, $\Gamma ^\sigma _\alpha$ can be expressed as, $\Gamma ^\sigma _\alpha = 2 \pi | t_{\alpha \sigma } |^2 \rho _{\alpha \sigma }$, with $\rho _{\alpha \sigma }$ being the constant spin-dependent density of states of the lead $\alpha$. By introducing the lead spin polarization $p_\alpha = (\rho _\alpha ^+ - \rho _\alpha ^-)/(\rho _\alpha ^+ + \rho _\alpha ^-)$, where $+$ $(-)$ corresponds to the majority (minority) spin species in the lead, the couplings can be written as $\Gamma ^\sigma _L = \Gamma _L (1 + \tilde{\sigma }p_L)$ and $\Gamma ^\sigma _R = \Gamma _R (1 \pm \tilde{\sigma }p_R)$, with $\Gamma _\alpha = (\Gamma _\alpha ^\uparrow + \Gamma _\alpha ^\downarrow )/2$. Here, $\tilde{\sigma }$ is equal to 1 (-1) for $\sigma = \uparrow$ ($\downarrow$) and the upper (lower) sign in $\Gamma ^\sigma _R$ corresponds to the parallel (antiparallel) magnetic configuration of the system. In other words, we assume that in the parallel configuration the spin-up electrons belong to the majority-spin subband of the leads. In the following, we assume that the couplings are symmetric, $\Gamma _L=\Gamma _R = \Gamma / 2$, and the leads are made of the same ferromagnetic material, $p_L = p_R\equiv p$.

Finally, the last part of the total Hamiltonian, $H_\mathrm{QD-Maj}$, describes the low-energy properties of quantum dot coupled to Majorana wire, and it can be expressed as^29,35,61^

2 $$\begin{aligned} H_\mathrm{QD-Maj} = \sum _{\sigma =\uparrow ,\downarrow } \varepsilon d^{\dag }_{\sigma } d_{\sigma } + U d^{\dag }_{\uparrow }d_{\uparrow } d^{\dag }_{\downarrow }d_{\downarrow } + V_M \left( d^{\dag }_\downarrow \gamma + \gamma d_\downarrow \right) + 2i\varepsilon _M \gamma \tilde{\gamma }. \end{aligned}$$

The quantum dot on-site energy is denoted by $\varepsilon$, while *U* is the Coulomb repulsion energy on the dot. The Majorana operator $\gamma$ ($\tilde{\gamma }$) describes the MZM near (far from) the quantum dot, whereas $V_M$ is the matrix element for hopping between the MZM and the quantum dot. We assume that $\gamma$ couples only to one spin species on the quantum dot, in particular, to spin-down electrons. The overlap between the MZMs is denoted by $\varepsilon _M$ and for long enough nanowires it can be neglected, $\varepsilon _M \rightarrow 0$. The Majorana operator $\gamma$ can also be expressed as a linear combination of auxiliary fermionic operators $\gamma = \left( f^\dag + f \right) /\sqrt{2}$, while the other Majorana mode, $\tilde{\gamma }= i\left( f^\dag - f \right) /\sqrt{2}$, remains decoupled from the dot. In a very general case the Majorana mode could couple to both spin components of the quantum dot, however, for the sake of clarity of the present analysis, we restrict ourselves to collinear alignments^29,61^. However, although in Eq. (2) the spin-down electrons couple to Majorana wire, to make the considerations more comprehensive, we also discuss the transport behavior in the case when the spin-up quantum dot level couples to the Majorana mode.

Let us start the analysis by presenting the eigenspectrum of the quantum dot-Majorana subsystem decoupled from the leads. For such system, one can easily find the eigenspectrum of $H_\mathrm{QD-Maj}$, $H_\mathrm{QD-Maj} |\chi \rangle = \varepsilon _\chi |\chi \rangle$, where we label the eigenstates by the number of spin-up electrons in the dot $n_{\uparrow }$, the states parity $P=e/o$ and sign ±, $|\chi \rangle \equiv |n_\uparrow ,P,\pm \rangle$. The corresponding eigenstates together with their eigenenergies are presented in Table 1. This eigenspectrum will be crucial in understanding the transport behavior at nonequilibrium settings, presented in the sequel, as the excitation energies between the even and odd states will be directly visible in the transport characteristics as a function of bias voltage and position of the orbital level, which can be effectively tuned with a gate voltage.

**Table 1:** Solving $H_\mathrm{QD-Maj}|\chi \rangle = \varepsilon _\chi |\chi \rangle$ gives rise to eight eigenstates presented in this table, $|\chi \rangle \equiv |n_\uparrow ,e/o,\pm \rangle$, where $n_\uparrow$ is the number of spin-up electrons, *e*/*o* refers to the electron parity (even or odd) in the subsystem.

Eigenenergy, $\varepsilon _\chi$	Eigenstate, $|n_\uparrow ,e/o,\pm \rangle$	$x=$
$( \varepsilon \mp \sqrt{x^2 + 2V_M^2} )/2$	$|0,o,\mp \rangle = \alpha (\mp x)|\downarrow 0\rangle + \alpha (\pm x)|0 1\rangle$	$\varepsilon - 2\varepsilon _M$
$( \varepsilon \mp \sqrt{x^2 + 2V_M^2} )/2$	$|0,e,\pm \rangle = \alpha (\pm x)|0 0\rangle - \alpha (\mp x)|\downarrow 1\rangle$	$\varepsilon + 2\varepsilon _M$
$( 3\varepsilon + U \mp \sqrt{x^2 + 2V_M^2} )/2$	$|1,o,\pm \rangle = \alpha (\pm x)|\uparrow 0\rangle - \alpha (\mp x)|d 1\rangle$	$\varepsilon + U + 2\varepsilon _M$
$( 3\varepsilon + U \mp \sqrt{x^2 + 2V_M^2} )/2$	$|1,e,\mp \rangle = \alpha (\mp x)|d 0\rangle + \alpha (\pm x)|\uparrow 1\rangle$	$\varepsilon + U - 2\varepsilon _M$

The coefficient $\alpha (\pm x)$ is defined as $\alpha (\pm x) = \sqrt{ 1 \pm x/\sqrt{x^2 + 2V_m^2}}$, with *x* specified in the table. The local states are $|\xi \eta \rangle$, where $\xi = \{0,\uparrow ,\downarrow ,d\}$ corresponds to empty, singly occupied with spin-up/spin-down electron or doubly occupied quantum dot, while $\eta = \{0,1\}$ is associated with the auxiliary operator *f*.

In the following, we will study the bias and gate voltage dependence of the transport coefficients considering two different cases of how the system is biased. In the first case, henceforth referred to as *symmetric* biasing case, the potential difference is applied between the ferromagnetic leads, which are assumed to share the same chemical potential ($\mu _L = \mu _R = eV$), and the Majorana wire ($\mu _S = 0$). On the other hand, in the second *asymmetric* case, the bias voltage is applied between the two ferromagnetic leads, $\mu _L = -\mu _R = eV/2$, while again $\mu _S = 0$, see also Fig. 1. In calculations we determine the currents flowing through the left and right ferromagnetic junctions, $I_L$ and $I_R$, while the current to Majorana wire can be found from $I_S = I_L + I_R$. All formulas used to determine the transport characteristics are presented in the “Methods” section.

**Figure 2:**
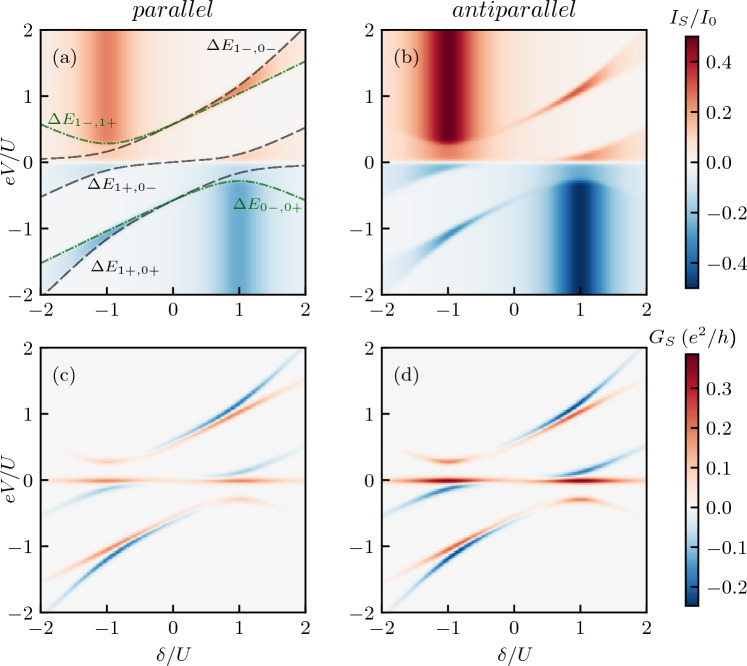
(**a**, **b**) The normalized current $I_S$ flowing between ferromagnetic leads and Majorana wire and (**c**, **d**) the corresponding differential conductance $G_S$ as a function of the bias voltage and quantum dot detuning $\delta =2\varepsilon +U$ for (left column) the parallel and (right column) antiparallel magnetic configuration of the device for symmetrically biased system, $\mu _L = \mu _R = eV$ and $\mu _S=0$. The dashed and dashed-dotted lines correspond to the excitation energies of the quantum dot-Majorana system, see the main text for details. Since the relevant single-particle excitations always happen between the even and odd parity states, we do not show the parity label here. The parameters are: $\Gamma = 0.01$, $k_BT=0.02$, $V_M = 0.2$, $\varepsilon _M = 0$, in units of $U \equiv 1$, and the lead spin polarization is $p=0.5$. The current is plotted in units of $I_0 = e\Gamma /\hslash$.

### The case of symmetrically biased system

In the case of symmetrically biased system the main interest is in the behavior of the current $I_S$ flowing between the ferromagnetic leads and Majorana nanowire. We will analyze the dependence of the current on magnetic configuration of ferromagnetic leads and also the resulting tunnel magnetoresistance (TMR) effect, which describes the change of the system transport properties when the magnetic configuration changes from the parallel to the antiparallel one. For the considered hybrid setup, we define the TMR as^62,63^

3 $$\begin{aligned} \text{TMR} = \frac{I^{P}- I^{AP}}{I^{AP}}, \end{aligned}$$

where $I^P$ and $I^{AP}$ designate the corresponding currents flowing in the parallel and antiparallel magnetic configuration, respectively.

#### Current and differential conductance

The normalized current $I_S$ together with the corresponding differential conductance $G_S=dI_S/dV$ is presented in Fig. 2 as a function of the bias voltage *eV* and the detuning from the particle-hole symmetry point $\delta = 2\varepsilon + U$. The left (right) column corresponds to the parallel (antiparallel) magnetic configuration of the system. First of all, one can note that the transport characteristics are antisymmetric with respect to the change of $\delta \rightarrow -\delta$ and $eV\rightarrow -eV$. Furthermore, both currents, $I_S^P$ and $I_S^{AP}$, exhibit qualitatively similar behavior in the full parameter space. This is also reflected in the differential conductance $G_S$ presented in the bottom row of Fig. 2. Because of this similarity, let us for the moment focus on the behavior of the current and conductance in the parallel configuration. We firstly observe a pronounced zero-bias anomaly in the differential conductance, which is a manifestation of the presence of Majorana zero-energy mode in the system. This anomaly extends over the whole range of the detuning parameter $\delta$, see Fig. 2c,d. With increasing the bias voltage, the current starts flowing and exhibits steps whenever the chemical potential of normal leads crosses the corresponding excitation energy of the Majorana-quantum dot subsystem. These excitation energies are marked in Fig. 2a with dashed and dashed-dotted lines and they actually coincide with the behavior of the differential conductance. We note that the possible excitations, due to single-electron tunneling, involve only the transitions between the even and odd parity states, therefore we suppress this index in the following notation. The black dashed lines marked by $\Delta E_{1\alpha ,0\beta }$ correspond to the excitation energies $\Delta E_{1\alpha ,0\beta } = \varepsilon + \left( U - \alpha \Lambda _U - \beta \Lambda \right) /2$, whereas the green dashed-dotted lines denoted by $\Delta E_{n_\uparrow -,n_\uparrow +}$ present the excitation energies $\Delta E_{1-,1+} = \Lambda _U$ and $\Delta E_{0-,0+} = -\Lambda$, with $\Lambda _U = \sqrt{(\varepsilon +U)^2+2V_M^2}$ and $\Lambda = \sqrt{\varepsilon ^2+2V_M^2}$. As can be seen in Fig. 2a,b, there are two regions of enhanced tunneling, one at $\delta \approx U$ for $eV < 0$ and another one at $\delta \approx -U$ for $eV > 0$. For those values of $\delta$ the current exhibits a large asymmetry with respect to the bias reversal. Consider the case of $\delta \approx -U$ for $eV > 0$. At equilibrium, the system is mostly occupied by the two states, which are degenerate, namely, $|1,o,+\rangle$ and $|1,e,-\rangle$. With increasing the bias voltage, around $eV\gtrsim \Delta E_{1-,1+} \approx U/4$, the other two states, $|1,o,-\rangle$ and $|1,e,+\rangle$, come into play giving rise to an increase in the current. These four states are equally occupied at larger voltages resulting in large current visible in Fig. 2a,b. However, once the bias voltage is reversed, the current becomes generally suppressed, except for the following regions of the bias voltage, $0\gtrsim eV \gtrsim \Delta E_{1+,0-}$ and $\Delta E_{0-,0+} \gtrsim eV \gtrsim \Delta E_{1+,0+}$, see Fig. 2a. Otherwise the system is trapped in certain states that block the current flow in the system. In particular, for $\Delta E_{1+,0-} \gtrsim eV \gtrsim \Delta E_{0-,0+}$, the system is occupied by the states $|0,o,-\rangle$ and $|0,e,+\rangle$, while for $eV \lesssim \Delta E_{1+,0+}$, it is occupied by the states $|0,e,-\rangle$ and $|0,o,+\rangle$. As a consequence, for negative bias direction one observes pronounced lines of negative differential conductance, see Fig. 2c,d. The mechanism for the current asymmetry with respect to bias reversal in the case of $\delta \approx U$ is similar to the one discussed above, and can be adequately adapted by performing the particle-hole transformation.

#### Tunnel magnetoresistance

**Figure 3:**
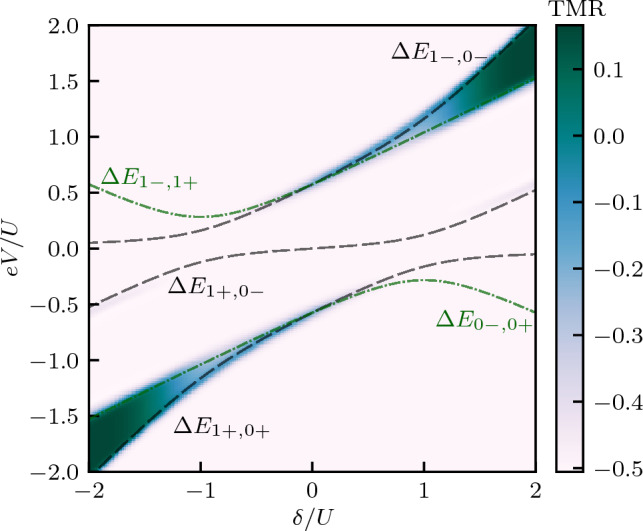
The tunnel magnetoresistance calculated as a function of the bias voltage *eV* and the detuning parameter $\delta$. The TMR is obtained from the currents in both magnetic configurations shown in Fig. 2. The dashed and dashed-dotted lines correspond to the excitation energies of the quantum dot-Majorana system, see the main text for details.

As can be seen in Fig. 2, a general tendency is that the current is larger in the antiparallel configuration compared to the parallel one. One can thus conclude that coupling to Majorana zero-energy mode facilitates transport through the system for the spin channel coupled to Majorana wire. The Majorana quasiparticle couples to the spin-down electrons on the dot, so for the parallel configuration this coincides with the minority-spin band of both leads, and therefore this enhancement is relatively weak. However, in the antiparallel configuration, the spin-down electrons always belong to majority band of one of the leads and, consequently, transport through one of the spin channels is enhanced, such that one finds $I_S^P < I_S^{AP}$ in most transport regions. This is clearly visible in Fig. 3, which presents the bias voltage *eV* and detuning $\delta$ dependence of the TMR. As can be seen, $\text{TMR } \approx -p$, except for the regions of bias voltage where $\Delta E_{1-,1+}\lesssim eV \lesssim \Delta E_{1-,0-}$, for $\delta \gtrsim -U$, and $\Delta E_{0-,0+}\gtrsim eV \gtrsim \Delta E_{1+,0+}$, for $\delta \lesssim U$, where one finds a weakly positive TMR. We also note that if one assumes that the Majorana mode couples to the spin-up electrons on the dot, the behavior of the TMR would be approximately reversed, with $\text{TMR}\approx p$ in most of the transport regimes (not shown).

#### Zero-bias anomaly

**Figure 4:**
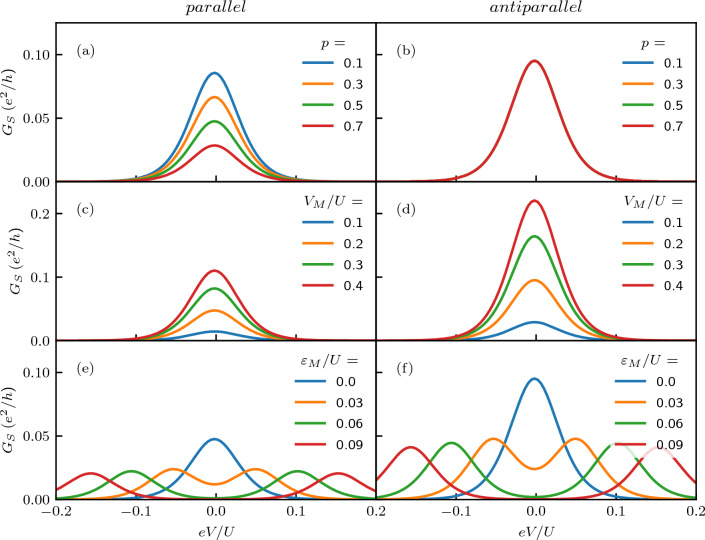
The dependence of the zero-bias anomaly in the differential conductance on (**a**, **b**) the degree of spin polarization of the leads for $V_M=0.2U$ and $\varepsilon _M=0$, (**c**, **d**) the strength of coupling to Majorana wire for $p=0.5$ and $\varepsilon _M=0$, and (**e**, **f**) the overlap between the two Majorana quasiparticles for $p=0.5$ and $V_M=0.2U$. The left (right) column presents the case of parallel (antiparallel) magnetic configuration. The other parameters are the same as in Fig. 2.

Let us now focus on the behavior of the zero-bias anomaly in the differential conductance due to the presence of Majorana zero-energy mode. Figure 4 presents the bias voltage dependence of the differential conductance $G_{S}$ for different values of spin polarization of ferromagnetic leads, for different values of strength of the coupling to Majorana wire $V_M$, and nonzero overlap $\varepsilon _M$ between the Majorana quasiparticles. First of all, one can see Fig. 4a that increasing *p* results in suppression of the zero-bias anomaly in the parallel configuration, while the anomaly does not depend on *p* in the antiparallel configuration, see Fig. 4b. This is because in the antiparallel configuration the relative number of spin-up and spin-down states available in the leads does not change with *p*, but is only transferred between the two leads. As far as the total current to both leads is concerned, this does not affect its magnitude, so antiparallel configuration effectively corresponds to the case of $p=0$. This is why for finite *p*, the anomaly is smaller in the case of parallel configuration compared to the antiparallel one. When the strength of coupling to Majorana wire is increased, it boosts the zero-bias anomaly, as can be seen in the second row of Fig. 4. This enhancement is clearly larger in the case of antiparallel configuration, which is due to the fact that conductance in this configuration behaves as in the $p=0$ case. Finally, the last row of Fig. 4 presents the bias dependence of the differential conductance for different values of the overlap between the Majorana quasiparticles $\varepsilon _M$. One can see that finite $\varepsilon _M$ results in the suppression of the conductance at the zero bias, while two satellite peaks, corresponding to split Majorana modes, emerge. The height of those peaks is approximately twice smaller than the peak for $\varepsilon _M = 0$ and this holds for the two magnetic configurations.

#### Current cross-correlations

**Figure 5:**
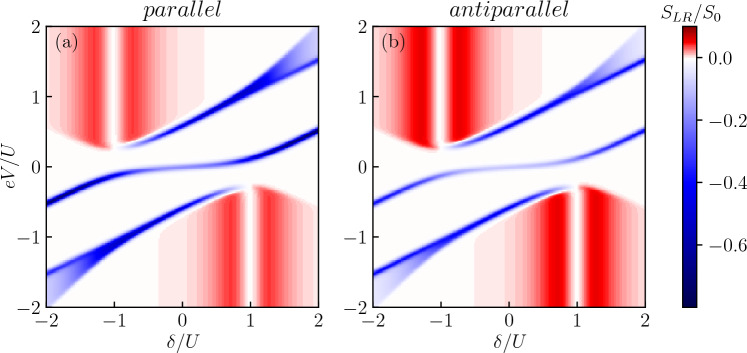
The current cross-correlations $S_{LR}$ calculated as a function of the bias voltage and detuning parameter $\delta$ for the case of (**a**) parallel and (**b**) antiparallel magnetic configuration of the system. The other parameters are the same as in Fig. 2 and $S_0 = e^2 \Gamma /\hslash$.

**Figure 6:**
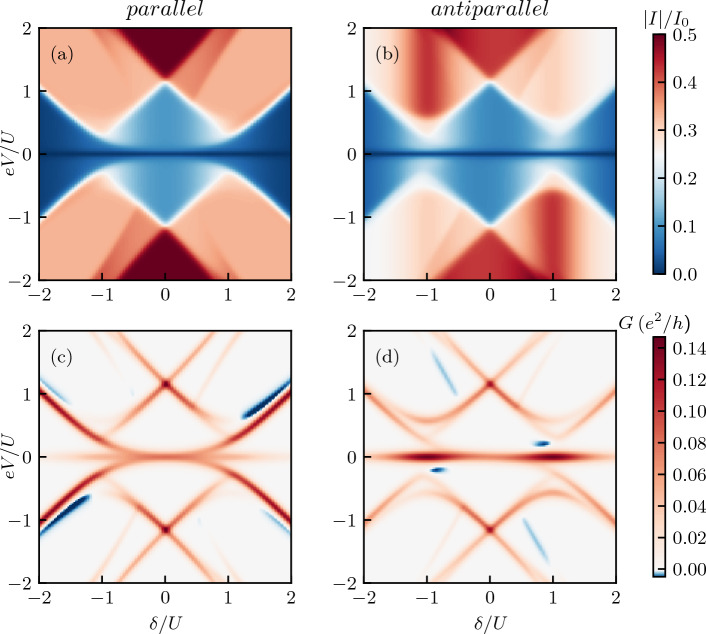
(**a**, **b**) The absolute value of the current $I\equiv I_R$ flowing to the right electrode and (**c**, **d**) the corresponding differential conductance $G=dI_R/dV$ as a function of the bias voltage and detuning $\delta$ for (left column) the parallel and (right column) antiparallel magnetic configuration of the device. The system is asymmetrically biased, $\mu _L = -\mu _R = eV/2$ and $\mu _S=0$. The other parameters are the same as in Fig. 2.

In the case of symmetrically biased device, when the current flows between the Majorana wire and two ferromagnetic contacts, further important information about the system transport properties can be obtained from the analysis of the cross-correlations between the left and right currents. Such cross-correlations calculated for the two magnetic configurations of the system are shown in Fig. 5. One can generally see that $S_{LR}$ takes large negative values only in certain narrow regimes of the bias voltage and detuning parameter. These regions actually coincide with the appropriate behavior of the current, cf. Fig. 2a,b. Large negative $S_{LR}$ indicates that the two currents are anti-correlated, i.e. on average a tunneling act through one junction is anti-correlated with a tunneling event through the other junction. Interestingly, for $\delta \approx -U$ and $eV>0$, and $\delta \approx U$ and $eV<0$, i.e. when we found maximum current, the cross-correlations are rather weak, with only small positive values. This implies that in these transport regions the currents through the left and right junctions are only very weakly correlated. We notice that positive cross-correlations were already predicted in quantum dot-Majorana systems, where non-local transport through both edges of the nanowire was present^64^. In our setup, however, such non-local processes are absent, and particles tunnel between one edge of the nanowire and single orbital of quantum dot coupled to metallic leads. It turns out that the crucial role here is played by the ratio $V_M/\Gamma$. When $V_M>\Gamma$, as considered in our analysis, the nanowire is a fast source of particles, while the bottleneck for transport is formed by the junctions between quantum dot and ferromagnets. Consequently, when a tunneling event takes place through one of those junctions, it is a process that immediately enhances probability of consecutive tunneling processes, since the quantum dot is quickly refilled with another particle in a fast process governed by $V_M$, which positively contributes to $S_{LR}$. This scenario takes place when the dot level is detuned from the resonance with the leads, while the left and right currents are not correlated ($S_{LR} = 0$) when the system is tuned to the resonant tunneling $\delta /U=\pm 1$, see Fig. 5. On the other hand, in the case of $V_M<\Gamma$, the system does not reveal positive cross-correlations anymore.

### The case of asymmetrically biased system

Let us now examine the case when the chemical potentials of the ferromagnetic leads are asymmetric, i.e. $\mu _L = -\mu _R = eV/2$, while the Majorana wire is grounded. In this case we focus on the current that flows out of the device to the right electrode, $I \equiv I_R$. Based on this current we explore the behavior of the differential conductance $G\equiv dI_R/dV$ and tunnel magnetoresistance, $\text{TMR} = I^P_R/I_R^{AP}-1$.

#### Current and differential conductance

The bias voltage and detuning dependence of the current and the differential conductance in both magnetic configurations of the device is shown in Fig. 6. The absolute value of the current clearly reveals the Coulomb diamond structure of the system. In the absence of Majorana mode, $V_M=0$, the current and differential conductance would exhibit a typical Coulomb diamond pattern. However, in the case of finite $V_M$, we observe a distortion of the Coulomb diamonds. First of all, the diamond lines do not cross at $\delta = \pm U$ when changing sign of the bias voltage, but there is an opening of the gap, see Fig. 6c,d. Moreover, a clearly visible zero-bias anomaly develops in the differential conductance, which indicates the leakage of Majorana quasiparticles onto the quantum dot system. In addition, there are also regions of very weak negative differential conductance.

#### Zero-bias anomaly

**Figure 7:**
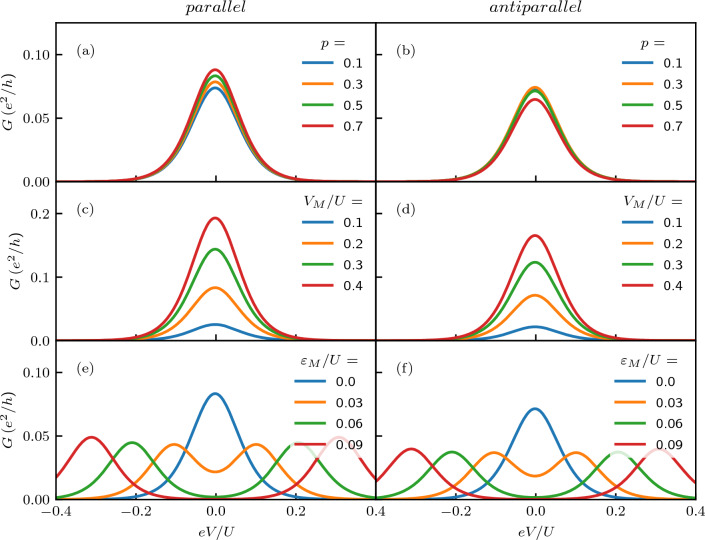
The dependence of the zero-bias anomaly in the differential conductance on (**a**, **b**) the degree of spin polarization of the leads for $V_M=0.2U$ and $\varepsilon _M=0$, (**c**, **d**) the strength of coupling to Majorana wire for $p=0.5$ and $\varepsilon _M=0$, and (**e**, **f**) the overlap between the two Majorana quasiparticles for $p=0.5$ and $V_M=0.2U$. The left (right) column presents the case of parallel (antiparallel) magnetic configuration. The other parameters are the same as in Fig. 6.

Let us now focus on the analysis of how the zero-bias anomaly depends on various system parameters, including lead spin polarization, strength of coupling to Majorana wire and the overlap between MZMs. The corresponding dependencies are shown in Fig. 7. One can see that, contrary to the symmetrically biased system, the conductance only very weakly depends on the spin polarization of the leads. While increasing *p* gives rise to a slight enhancement of *G* in the case of parallel configuration, it results in a small suppression of *G* for the antiparallel case. In both magnetic configurations increasing $V_M$ leads to comparable enhancement of the zero-bias anomaly. Finally, for shorter wires, when finite overlap $\varepsilon _M$ emerges, the anomaly becomes split and only two side peaks are present, see the last row of Fig. 7.

**Figure 8:**
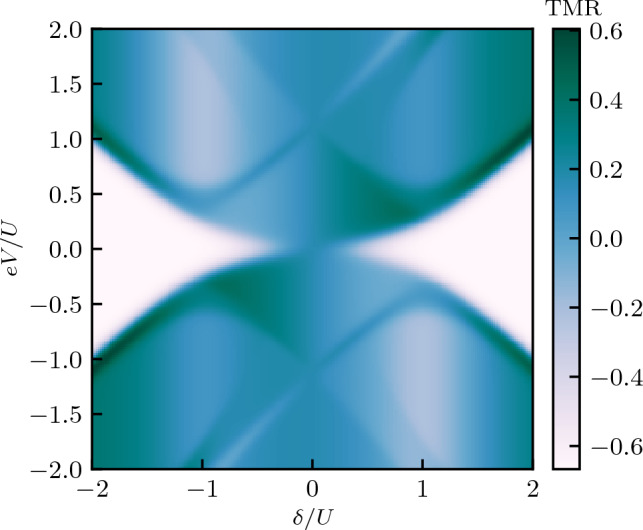
The tunnel magnetoresistance calculated as a function of the bias voltage *eV* and the detuning parameter $\delta$. The other parameters are the same as in Fig. 6.

**Figure 9:**
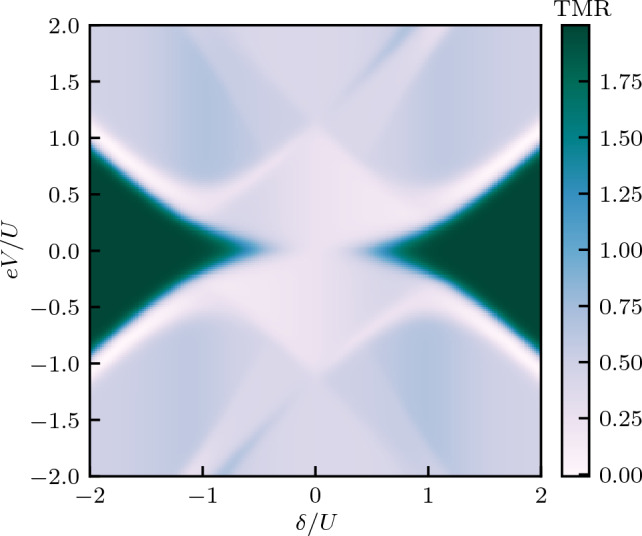
The same as in Fig. 8, but calculated for the case when the Majorana mode couples to the spin-up electrons in the quantum dot.

#### Tunnel magnetoresistance

The difference in the currents in the two magnetic configurations gives rise to finite TMR, which is presented in Fig. 8. As can be seen, now, the dependence of the TMR is much more complex, with pronounced regions of both positive and negative TMR. This is due to the fact that TMR is obtained based on the current that flows out of the device to the right ferromagnetic lead. Such setup resembles thus a ferromagnetic tunnel junction, however, with a complex quantum dot-Majorana nanostructure embedded in the junction. In the absence of this nanostructure, the TMR would be given by the Julliere value^62^, $\text{TMR} = 2p^2/(1-p^2)$, which for assumed spin polarization ($p=0.5$) yields, $\text{TMR} = 2/3$. Furthermore, in the case of embedded quantum dot (the case of $V_M=0$), the TMR would get reduced and would range from $\text{TMR} = p^2/(1-p^2)$ ($\text{TMR}=1/3$) to $\text{TMR} = (4/3)p^2/(1-p^2)$ ($\text{TMR}=4/9$)^65^. Interestingly, in the presence of Majorana quasiparticles, we find a greatly modified TMR pattern. At the onset of steps in the current, the TMR is very much suppressed and approximately given by $\text{TMR}\approx 0$. For larger voltages, however, the TMR becomes positive but generally lower than the Julliere TMR. On the other hand, a negative tunnel magnetoresistance is found at low bias voltages for $|\delta | \gtrsim U/2$, where the absolute value of TMR is approximately given by the Julliere value (for $p=0.5$), see Fig. 8.

Let us also comment on the case when the Majorana mode is coupled to the spin-up electrons in the quantum dot. Such change of coupling is most visible in the behavior of the TMR, which for this case is presented in Fig. 9. Now, opposite to the previously-discussed situations, the TMR is positive in the whole parameter space. For larger voltages, there is an enhancement of the TMR to around the Julliere value. Moreover, as can be seen in Fig. 9, a largely increased tunnel magnetoresistance is found at low bias voltages for $|\delta | \gtrsim U/2$, where the TMR exceeds by a few times the Julliere value. Such high values of the TMR are associated with the presence of MZM in the system.

## Discussion

As follows from the above presented results, the presence of Majorana zero-energy modes gives rise to some unique spin-resolved transport properties of the system, visible in the current, differential conductance, tunnel magnetoresistance, as well as the current cross-correlations. We have in particular studied the bias voltage and orbital level detuning dependence of those quantities for two different ways in which the system is biased.

In the firstly studied case of symmetric biasing, we have examined the transport between the ferromagnetic leads, kept at the same chemical potential, and the grounded Majorana wire. We have identified regions of negative differential conductance, which can be explained by invoking the corresponding excitation energies of quantum dot-Majorana subsystem. We have also analyzed the bias-reversal asymmetric dependence of the current, where the suppression of tunneling was associated with trapping the system in certain states. The difference in transport properties when the magnetic configuration of the device is varied was captured by the tunnel magnetoresistance. The TMR for assumed model parameters turned out to be mostly negative, $\text{TMR}\approx -p$, with narrow regions of positive TMR in the parameter space of *eV* and $\delta$. For the symmetrically biased system, we have also determined the current-current correlation function, which revealed regions of both positive and negative values, indicating the type of cross-correlations between the currents flowing through the ferromagnetic junctions. A special attention has been paid to the dependence of the zero-bias anomaly on the system’s magnetic configuration and the degree of spin polarization of the leads. We have shown that the conductance does not depend on spin polarization in the case of antiparallel configuration, whereas for parallel system’s configuration, increasing the lead spin polarization results in suppression of the zero-bias anomaly. Moreover, we have also examined the effect of finite length of the Majorana nanowire and showed that with increasing the overlap of Majorana quasiparticles $\varepsilon _M$, the zero-bias anomaly becomes suppressed and the differential conductance exhibits only side resonances at voltages corresponding approximately to $\pm \varepsilon _M$.

On the other hand, for the asymmetrically biased device, we have analyzed the dependence of the current, differential conductance and TMR associated with the current flowing through the right junction. We have shown that the presence of Majorana quasiparticles modifies the typical Coulomb diamonds and gives rise to zero-bias anomaly. Moreover, even more pronounced signatures are observed in the tunnel magnetoresistance, which exhibits negative or positive values depending on the bias and gate voltages. In fact, in certain transport regions we found the TMR by several times exceeding the TMR of the system consisting of a bare quantum dot coupled to ferromagnetic leads.

Our study thus reveals new features observable in spin-dependent transport properties of the Majorana-quantum dot system that may serve as further indications of the presence of Majorana quasiparticles in the system. Moreover, by providing a comprehensive analysis of spin effects on the nonlinear transport properties of the considered system, our work promotes further investigations of quantum dot-Majorana wire devices. Finally, as an outlook, we would like to mention that further insight into the behavior of such hybrid systems can be obtained from the studies of thermoelectric transport, where e.g. sign changes of the thermopower can provide additional information about the presence of Majorana zero-energy modes in the system^25,33,66–69^.

## Methods

We determine the spin-dependent transport properties of the system by using the real-time diagrammatic technique^56–58,70^. This approach consists in a systematic perturbation expansion of the quantities of interest with respect to the coupling strength $\Gamma$ to the ferromagnetic leads. Within the real-time diagrammatic technique, the time evolution of the system’s reduced density matrix can be visualized as a sequence of irreducible diagrams on the Keldysh contour^56,70^. On the other hand, the elements of the reduced density matrix can be found from a general kinetic equation within Markov approximation by including diagrams of given order, which can be found from the corresponding diagrammatic rules^56–58,70^. Our interest lies in the sequential tunneling regime, where transport is determined through one-by-one electron tunneling processes. The current flowing between the ferromagnetic leads and the Majorana wire can be found from the Kirchhoff’s law, $I_S = I_L + I_R$. Here, $I_\alpha$ is the current flowing through the $\alpha$-junction with the normal lead, which can be found from

4 $$\begin{aligned} I_\alpha = \frac{e}{2\hslash }\text{Tr} \{ \mathbf {W^{I_\alpha } p^\text{st}} \}, \end{aligned}$$

where $\mathbf {p^\text{st}}$ denotes the stationary occupation probability vector, the elements of which $p^{st}_{\chi }$ describe the probability of occupying the eigenstate $|\chi \rangle$. The probabilities can be in turn calculated from the following kinetic equation

5 $$\begin{aligned} \mathbf {W p^\text{st} } = 0, \end{aligned}$$

by using the normalization condition $\sum _\chi p^{st}_\chi = 1$. The elements of the self-energy matrix, $W_{\chi \chi '}$, correspond to transitions between the states $|\chi \rangle$ and $|\chi '\rangle$, while $\mathbf {W^{I_\alpha }}$ is similar to $\textbf{W}$ but it takes into account the number of particles transferred through the junction $\alpha$. The self-energies can be found by evaluating contributions from first-order diagrams that are topologically different by using the diagrammatic rules^56–58^.

Finally, the diagrammatic expression for current cross-correlations between the left and right junctions in the first-order approximation is given by^57,58^

6 $$\begin{aligned} S_{LR}=\frac{e}{\hslash }\text{Tr}\{(\mathbf {W^{I_L}PW^{I_R}+W^{I_R}PW^{I_L})p^\text{st}} \}, \end{aligned}$$

where $\textbf{P}$ denotes the corresponding propagator.

## Data Availability

The datasets generated and analyzed during the current study are available from the corresponding author on reasonable request.
